# Proceedings of the Chicago Medical Society

**Published:** 1884-10-20

**Authors:** 


### PROCEEDINGS OF THE CHICAGO MEDICAL SOCIETY.

At the regular semi-monthly meeting of this Society, held September 25th, 1884, Dr. C. W. Earle presented an interesting paper on "Malformation of the Stomach," and exhibited a specimen (the stomach) of a new-born babe that lived twelve days. There was no opening from the stomach leading into the duodenum. Biliary matter was found in the contents of the bowels and passages. The same gentleman also presented the history of a case of bony tumor of the female pelvis, and exhibited the pathological specimen, which weighed three and a half pounds. Bony tumors of this region are rarely seen, we think.

The Secretary, Dr. Liston H. Montgomery, offered the following resolutions and moved their adoption:

Whereas, From present reports and indications in foreign countries, cholera and yellow fever (both pestilential diseases) prevail, and as the latter especially is always assuming a threatening attitude toward us, and is not conducive to our national prosperity, nor to public health, and should, if possible, be averted with earnest and efficient sanitary measures; and

Whereas, Cholera may make its appearance on this continent ere another twelve months should elapse, and should likewise, if possible, be averted or restricted to the narrowest limits; therefore,

Resolved, That it is the sense of the Chicago Medical Society to have that department of the government relating to public health recognize the services of able sanitarians who constitute the National Board of Health, for the purpose of co-operating with municipal, State and other organizations of a similar kind, and that a committee of seven (7) members of this Society be appointed by the Chair to draft suitable resolutions in behalf of said National Board.

Resolved furthermore, That this Committee present said resolutions to the Congress of the United States, memorializing that body to make a sufficient appropriation for the purpose of said Board for scientific investigation in the prevention and restriction of epidemic, preventable, and pestilential diseases.

We believe this action should be promptly taken at the coming session of our national legislature, and that a thorough sanitary organization of the nation should be recognized, and with it absolute enforcement of the best means for the protection of her citizens and the improvement of our inter-State sanitary condition.

The resolutions, as read, provoked a good deal of discussion, which was participated in by many of the most influential members of the Society, all of whom favored their adoption. The motion to this effect was unanimously carried. Dr. J. H. Etheridge moved that the committee be authorized to present their resolutions first to the Society for a final consideration before they are presented to Congress, which motion also prevailed.

The following well-known members constitute the committee: Drs. O. C. DeWolf, R. E. Starkweather, L. H. Montgomery, John Bartlett, J. H. Etheridge, A. R. Jackson, J. H. Hollister.

